# Effect of Preformed Polymeric Microspheres on the Frost Resistance of Low-Clinker Cementitious Composites with Fine Recycled Aggregate

**DOI:** 10.3390/ma18235438

**Published:** 2025-12-02

**Authors:** Maja Kępniak, Rafał Panek, Maciej Kalinowski, Wojciech Franus

**Affiliations:** 1 Department of Building Materials Engineering, Faculty of Civil Engineering, Warsaw University of Technology, Al. Armii Ludowej 16, 00-637 Warsaw, Poland; maciej.kalinowski@pw.edu.pl; 2 Department of Construction Materials Engineering and Geoengineering, Lublin University of Technology, 40 Nadbystrzycka Str., 20-618 Lublin, Poland; r.panek@pollub.pl (R.P.); w.franus@pollub.pl (W.F.)

**Keywords:** low-clinker cement, polymeric microspheres, freeze–thaw resistance, air–void structure, fine recycled aggregate

## Abstract

Achieving adequate frost resistance in cementitious composites made with low-clinker binders remains challenging, as conventional air-entraining admixtures often show limited effectiveness in such systems. This study examines an alternative approach that involves incorporating preformed polymeric microspheres to create a stable air–void system and enhance freeze–thaw durability. Cementitious composites were prepared using a low-clinker binder containing fly ash and ground granulated blast furnace slag (GGBFS) as supplementary cementitious materials, with natural sand partially replaced by fine recycled aggregate derived from concrete waste. The influence of polymeric microspheres on workability, compressive strength, pore structure, and frost resistance was evaluated. Compared to the reference mixture (32.8 MPa), the mortar modified with polymeric microspheres exhibited clearly higher compressive strength—about 25% greater after 28 days—while the AEA-modified mixture showed a slight reduction. Total porosity measured by MIP was 18% for REF, 19% for AEA, and 17% for PPMThe results showed that adding polymeric spheres initially introduced a network of discrete voids that improved the material’s resistance to early freeze–thaw cycles. However, due to the prolonged hydration of the low-clinker system, hydration products progressively filled the initially created voids after the partial degradation of the polymeric spheres. Consequently, the air–void system gradually disappeared, leading to a loss of frost resistance at later ages. After 100 cycles, the PPM mixture exhibited a 75% loss in flexural strength and a 35% loss in compressive strength, whereas the AEA mixture retained its durability, with compressive strength loss limited to 6%. This finding suggests that, although early tests may indicate improved performance, the long-term durability of low-clinker cementitious composites incorporating fine recycled aggregate cannot be reliably enhanced by preformed polymeric spheres alone.

## 1. Introduction

Durability is one of the key factors that must be considered in the design of sustainable cementitious composites [1]. One of the most aggressive environmental factors affecting cement-based materials in many regions is cyclic freezing and thawing [2,3]. Designing freeze–thaw-resistant composites that simultaneously meet sustainability requirements is particularly challenging, as it requires careful control of composition [4,5], microstructure [6,7], and pore distribution [7,8]. Among these factors, the impact of the porosity characteristics of the cement matrix is critical [9,10]. An excessive pore network increases water absorption and promotes freeze–thaw damage. In contrast, a well-distributed system of discrete, stable air voids can mitigate internal stress caused by the phase change of water.

Issues regarding durability become especially important when using low-clinker cement, which are developed to reduce the carbon footprint of cement-based materials. Such binders typically contain high amounts of supplementary cementitious materials (SCMs), such as fly ash and ground granulated blast furnace slag (GGBFS). While these additions improve sustainability, they also slow down hydration and alter the pore structure, often resulting in lower early strength and reduced frost resistance. While these additions improve the composite’s sustainability, they also alter the hydration process, prolonging it and, consequently, changing the pore structure of the matrix, which often results in lower early strength and reduced durability. However, in systems containing fly ash, air-entraining admixtures (AEAs) may be adsorbed onto the surface of ash particles, especially unburnt carbon, reducing their effectiveness [11,12,13]. This phenomenon can require AEA dosages several times higher than recommended to achieve the desired air–void system [11,14,15], which increases variability and complicates mixture design. Furthermore, stable, long-term air–void systems are challenging to ensure due to the ongoing hydration of the binder, which gradually refines the pore structure.

The residual (unburnt) carbon present in fly ash can significantly interfere with the action of air-entraining admixtures (AEAs) [16,17]. First, the porous carbon particles provide a large specific surface area that adsorbs surfactant molecules, reducing their availability in the paste. Second, the effective dosage of AEA is thereby reduced and must be increased to achieve the target air contents. Third, the destabilization of the air–void system occurs because fewer stabilized microbubbles are formed, leading to the coalescence or collapse of voids. Finally, increased air loss during mixing results from this destabilized void system and surfactant loss to carbon surfaces. Such mechanisms together explain why fly ash with an elevated unburnt carbon content often requires higher AEA dosages and exhibits less reliable freeze–thaw durability.

In response to these challenges, recent research has explored the use of preformed polymeric microspheres as an alternative to AEAs for improving the frost resistance of concrete [18,19]. Unlike AEAs, which chemically generate air bubbles of varying sizes and often result in inconsistent spacing and significant strength loss (typically a 5% strength reduction per 1% of increase in entrained air volume), polymeric microspheres introduce rigid, uniformly sized voids without relying on chemical foaming mechanisms. These microspheres are typically 10–100 µm in diameter, hydrophobic, and dimensionally stable over time [20]. When incorporated at very low dosages (0.7–1.0% by volume of concrete, corresponding to approximately 0.015–0.025% by cement mass), they create a fine and evenly distributed air–void system that requires only about 0.26–0.7% of entrained air to provide adequate freeze–thaw protection-compared to 4–6% required for conventional AEA-based systems [21]. The frost protection mechanism of polymeric microspheres is primarily physical. During freezing, their higher thermal expansion coefficient relative to the cement matrix leads to slight contraction, forming annular gaps at the interface between the spheres and the hardened paste [22]. These gaps act as micro pressure-relief sites, preventing internal cracking caused by ice formation. This results in a highly refined air–void structure with low spacing factors (typically 0.06–0.12 mm), which meets or exceeds standard durability requirements [23]. In both laboratory and field studies, such systems have demonstrated freeze–thaw and salt-scaling resistance equivalent or superior to AEA-modified concretes, while maintaining higher compressive strength due to the lower total air content and showing stable void structure during long-term exposure [21,24].

The issue of porosity in concrete becomes even more complex when incorporating recycled aggregates, as it involves not only the new cement matrix but also the micro-pores and micro-cracking inherent within the recycled aggregate particles themselves [10]. This dual-level porosity, both in the matrix and within the aggregates, creates unique challenges in terms of controlling the overall pore structure [25] and enhancing the material’s resistance to freeze–thaw cycles [9]. Additionally, the Interfacial Transition Zone (ITZ) between the recycled aggregate and the cement matrix behaves differently from that of conventional concrete. In recycled concrete, the ITZ is typically weaker, resulting in reduced bonding strength and altered microstructural behavior, which can influence the material’s performance in freeze–thaw conditions [26]. The frost resistance of recycled fine aggregate concrete deteriorates with decreasing particle size and increasing content of recycled fine aggregate, as smaller particles increase surface area, leading to higher water absorption and an increased risk of freeze–thaw damage [27].

Therefore, by incorporating preformed polymeric spheres, this study evaluates the possibility of achieving frost resistance in cementitious composites that incorporate low-clinker cement (containing fly ash and ground granulated blast-furnace slag, or GGBFS) and fine recycled aggregate (FRA). The research investigates the effect of these spheres on the fresh properties, compressive strength, porosity, microstructure, and freeze–thaw durability of the composites. Particular attention is paid to the long-term development of porosity, considering the slower hydration rate typical of low-clinker binders, which may affect the stability of the preformed voids.

## 2. Materials and Methods

This study was carried out on cement mortars based on a low-clinker cement containing fly ash and ground-granulated blast-furnace slag. The modification under investigation was an admixture of preformed polymeric microspheres used as pore formers (PPM). The mortars are prepared with a constant 15% sand substitution with fine recycled aggregate. Two reference mortars were also prepared: a plain mortar without any air-entraining component (REF) and a mortar with a conventional air-entraining admixture (AEA).

To ensure uniform dispersion, the microspheres (or, for reference, the AEA) were first introduced into the mixing water and dispersed. Next, cement was added and mixed to obtain a homogeneous paste. Finally, the aggregate was incorporated, and mixing continued until a uniform mortar was obtained.

The experimental program evaluated the impact of admixtures on consistency (after mixing), flexural and compressive strength, freeze–thaw resistance, porosity, and microstructure (using image-based analysis). Detailed mixture proportions, dosages, specimen preparation, curing, and test procedures are provided in the following subsections.

### 2.1. Materials

#### 2.1.1. Cement

A multicomponent low-clinker cement classified as CEM V/A (S-V) 42.5 N-LH/HSR/NA (Holcim, Kujawy, Poland) was used as the binder. The cement complies with PN-EN 197-1 [28] and PN-B-19707 [29] requirements and contains granulated blast furnace slag (S) and siliceous fly ash (V) as the main supplementary cementitious constituents. The cement is characterized by normal early strength (42.5 N), high resistance to sulfate attack (HSR), low heat of hydration (LH), and low water-soluble alkali content (NA). It has a Blaine fineness of approximately 4900 cm^2^/g, an initial setting time of 270 min, and compressive strengths of about 20.5 MPa after 2 days and 57.7 MPa after 28 days. The environmental product declaration (EPD) prepared in accordance with EN 15804+A2 [30] and ISO 14025 [31] reports a global warming potential (GWP) of 323 kg CO_2_-eq per 1 Mg of cement for stages A1–A3 (cradle-to-gate).

#### 2.1.2. River Sand and Fine Recycled Aggregate

**Figure 1:**
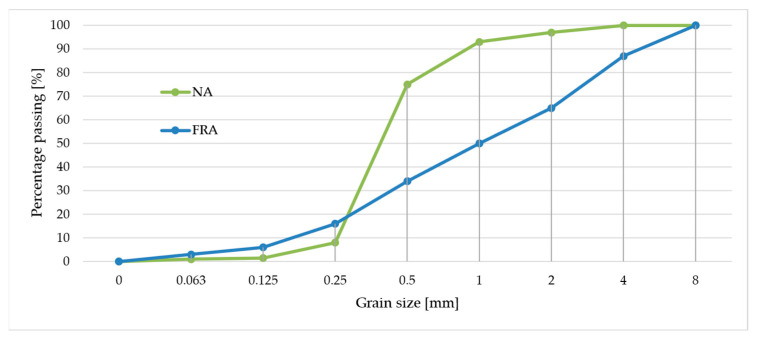
The grain size distribution of sand (0/2) used in the study.

A river sand (NA) with particle size up to 2 mm (0/2 fraction) sourced from the Vistula River was used for the fine aggregate. This sand conforms to the following standard requirements: PN-EN 933-1 [32] for particle size distribution (gradation), PN-B-06714-46 [33] regarding alkali-silica reaction potential, and PN-EN 1744-1 [34] concerning organic contaminants. The gradation curve of the sand was determined, showing a well-graded distribution within the 0/2 mm range (detailed sieve analysis results are presented in Figure 1). This sand was chosen due to its local availability, compatibility with cementitious systems, and compliance with standard requirements for fine aggregates in mortar and concrete applications.

**Figure 2:**
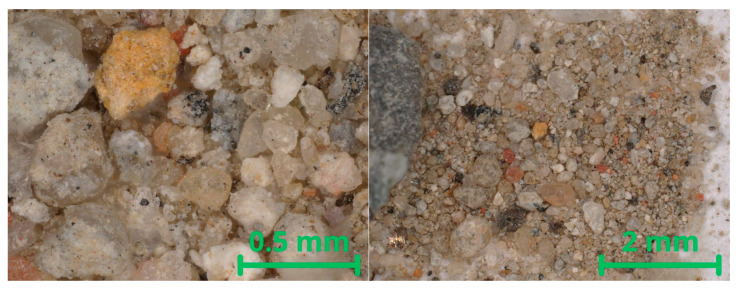
Optical microscopic image of the recycled fine aggregate used in the study (Keyence).

The fine recycled aggregate (FRA) used in this study was obtained from the crushing and subsequent screening of construction and demolition waste (C&DW) from an approximately 30-year-old reinforced concrete residential building in Poland. Due to its origin from absolute demolition waste, the material was heterogeneous and not composed exclusively of concrete grains. Optical microscopy observations confirmed the presence of particles with diverse surface textures and colors, indicating a composition similar to the coarse recycled aggregate produced in the same crushing batch (Figure 2).

The composition of the coarse fraction from this batch was previously analyzed according to PN-EN 933-11 [32] and was used as a reference for the likely composition of the recycled sand. The results were as follows: Rc (concrete, mortar, and concrete products)—88.5%, Ru (unbound aggregate, natural stone, hydraulically bound aggregate)—4.7%, Rb (clay, silicate, cellular concrete)—0.2%, Ra (bituminous materials)—1.9%, and X (other materials such as metals, wood, plastic, rubber, gypsum plaster)—4.5%.

The apparent density of the recycled sand grains was 2.59 g/cm^3^, and their water absorption was 8.2%. The grain size distribution of the recycled sand is presented in Figure 1.

#### 2.1.3. AEA

As a reference admixture, a commercial air-entraining agent (CHRYSO^®^Air LB, CHRYSO Polska Sp. z o.o., Warsaw, Poland) conforming to EN 934-2:2009+A1:2012 [35] was used. It is a water-soluble liquid with a density of approximately 1.00 kg/dm^3^, pH ≈ 7, and contains less than 0.1% chlorides and less than 0.3% alkalis (as Na_2_O equivalent).

The admixture contributes to increased water retention and reduced segregation and bleeding in fresh mixtures. The admixture was introduced into the mixing water to promote uniform dispersion before cement addition. The recommended dosage is approximately 0.2% by cement mass, with an allowable range from 0.04 to 1.5 kg per 100 kg of cement.

#### 2.1.4. Polymeric Microspheres Admixture

**Figure 3:**
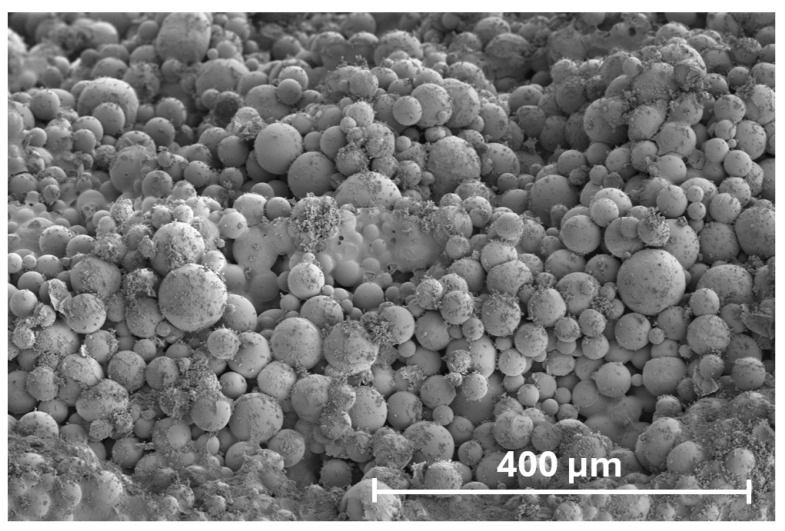
SEM micrography of the spherical morphology of polymeric microspheres used in the study.

Prefabricated, hollow polymer microspheres (mean diameter 47.3 µm ±20% tolerance, bulk density of 0.102 g/cm^3^) of a spherical morphology (Figure 3) were used as a physical air-entraining admixture (Centrament AirPolymer, MC-Bauchemie, Bottrop, Germany). Because no detailed grain size distribution is provided, the tolerance range (37.8–56.8 µm) was treated as the ±2σ interval of a normal distribution. This yields a mean value of μ = 47.3 µm and a standard deviation of σ ≈ 4.73 µm, with characteristic percentiles of D10 ≈ 41.2 µm and D90 ≈ 53.4 µm. When added to the concrete matrix, they introduce a controlled population of voids with a defined size distribution, based on the mass of admixture introduced to the mix during the composite’s preparation. The particles remain intact during mixing, transport, and placement within the cement matrix. After matrix hardening, these voids serve as local stress-relief reservoirs that accommodate the volumetric expansion of pore water during freezing, thereby reducing internal tensile stresses and limiting microcracking under freeze–thaw loading. The recommended dosage is up to 7 kg per 1 m^3^ of mix.

#### 2.1.5. Mortar Composition

The mixtures were designed to directly compare the influence of different air-entraining strategies on the properties of mortars containing recycled fine aggregate. Three mortar variants were prepared: a reference mixture (REF) without any air-entraining component, a mixture with a conventional air-entraining admixture (AEA), and a mixture with preformed polymeric microspheres (PPM).

A constant binder content, water-to-cement ratio, recycled aggregate, and total aggregate volume were maintained for all mixtures designed in the study, with only the type of air-entraining component varying between the series. The mass-based mixture compositions are summarized in Table 1.

To ensure a homogeneous distribution of polymeric microspheres within the mortar, the microspheres were first pre-dispersed in the mixing water and introduced at the initial stage of the mixing process. This procedure enabled the immediate wetting of the spheres by the cement paste, while the viscosity of the fresh mixture effectively counteracted the low bulk density of the spheres, preventing flotation. The mixing process was conducted without interruptions, limiting the risk of segregation. Density measurements of hardened prisms confirmed the absence of stratification, indicating a uniform distribution of the polymeric microspheres throughout the material.

### 2.2. Methods

#### 2.2.1. Consistency and Density

The consistency of the fresh mixtures was determined after 5 min, 60 min, and 90 min of mixing, following the procedure specified in PN-EN 1015-3 [36] for assessing the plasticity of construction mortars. The test was carried out using a flow table. A conical mold with a base diameter of 100 mm, a top diameter of 70 mm, and a height of 60 mm was filled with fresh mortar and lifted vertically. The table was subsequently subjected to 15 drops from a height of 10 mm at a frequency of one drop per second. After completing the jolting sequence, the final spread diameter of the mortar was measured as an indicator of consistency.

The volume density of the fresh mortar was determined immediately after mixing to evaluate the influence of individual admixtures on mixture compaction and entrapped air content. Additionally, the volume density of hardened specimens was measured after 28 and 56 days of curing, providing insight into the evolution of the internal structure and the degree of hydration over time.

#### 2.2.2. Flexural and Compressive Strength

The flexural strength was determined on three prismatic specimens (40 mm × 40 mm × 160 mm) prepared from each mortar composition, following the procedure specified in EN 196-1 [37].

All specimens intended for mechanical testing were cured under controlled conditions. Immediately after casting, the molds were covered with a polyethylene foil and stored for 24 h at laboratory temperature (20 ± 2 °C). After demoulding, the specimens were submerged in water at 20 ± 1 °C for the following 27 days or 57 days. Mechanical tests were performed immediately after removal from the curing water, without any additional surface drying.

The tests were carried out using a three-point bending configuration with a loading rate of 50 N/s. After the flexural tests, the two halves of each broken prism were used to determine the compressive strength, resulting in six specimens per series. The compressive tests were performed in accordance with EN 196-1 [37] on specimens with a loading surface area of 1600 mm^2^, using a loading rate of 2400 N/s. Both flexural and compressive strength values were recorded with an accuracy of 0.1 MPa.

#### 2.2.3. Freeze-Frost Resistance

The freeze–thaw resistance of mortar composites was evaluated according to PN-B-06265, which specifies cyclic freezing in air followed by thawing in water. This method was selected to assess the potential use of these mixes, which combine fine recycled aggregate and air-entraining components, in future concrete applications where resistance to extended freeze–thaw exposure is required.

After demolding, the specimens were cured for 24 h under foil in laboratory conditions and subsequently for 27 days in water. Before testing, the specimens designated for freeze–thaw exposure were saturated with water, surface-dried, weighed, and then placed in the freezing chamber. Each cycle consisted of 4 h of freezing in air at −18 ± 2 °C, followed by 4 h of thawing in water at +18 ± 2 °C, resulting in a total cycle time of 8 h.

Two exposure levels were applied: 25 cycles, corresponding to the requirement for mortars, and 100 cycles, corresponding to the requirement for concretes. After each exposure level, the mechanical properties were compared with reference samples stored in water (witness specimens) for the entire test period.

Three specimens (40 × 40 × 160 mm) were tested for each mixture and exposure level. Each prism was first subjected to a three-point flexural test, after which the two halves were used for compressive strength tests, resulting in three flexural strength and six compressive strength values for both the freeze–thaw series and the corresponding reference series.

#### 2.2.4. Porosity

Mercury intrusion porosimetry (MIP) was used to assess the influence of admixtures on the pore-network characteristics of cementitious composites. Measurements were conducted in intrusion mode on a Poremaster 33 (Quantachrome Instruments, Inc., Boynton Beach, FL, USA). The technique yields quantitative determinations of cumulative and differential pore size distributions, total mercury-accessible porosity, and information on pore connectivity by forcing non-wetting mercury into the pore system under progressively increasing pressure. Specimens for MIP were cured for 56 days under the same procedure as the samples for other tests and prepared by mechanically sectioning prismatic samples (40 × 40 × 10 mm) from the mid-length region of 40 × 40 × 160 mm samples to ensure representativeness with respect to the samples used for other tests. Immediately after cutting, specimens were cleaned in an ultrasonic bath with deionized water to remove surface fines and were subsequently dried at 105 °C for 48 h to eliminate residual moisture that could compromise mercury pressure/volume measurements. After cooling, the dry mass of each specimen was recorded, and the sample was gently comminuted to fit and fill approximately 75% of the volume of the penetrometer cell. Crushing was performed carefully to minimize preparation-induced damage and the creation of artificial microcracks in the sample.

MIP tests combined the instrument’s low- and high-pressure stations to span a broad pore diameter range of the cement matrix from approx. 110 µm down to 0.0064 µm. The low-pressure station accessed a larger pore content (approx. 2–20 psi; ~13.8 kPa to 138 kPa), while the high-pressure sequence extended the applied pressure from 20 psi to 33,000 psi (approx. 138 kPa to 227.5 MPa), enabling characterization of smaller pore diameters. As the pressure increased, the incremental and cumulative volumes of intruded mercury were recorded.

The specimen bulk volume was derived from the penetrometer geometry and the recorded tare, allowing for the conversion of intruded mercury volume into porosity and solid-phase specific and apparent densities. Conversion of pressure to an equivalent pore radius was performed using the Washburn equation (Equation (1)), where r is the pore radius (m), γ is the mercury-air surface tension (N·m^−1^), θ is the contact angle between mercury and the solid surface (rad), and P is the applied capillary pressure (Pa). Incremental intrusion data were used to determine the differential and cumulative pore distributions, as well as summary metrics.

(1) $$r=-\frac{2\gamma cosϴ}{P}$$

Pore-network morphology was quantified using two parameters: the surface fractal dimension (D) and the pore tortuosity (τ). The fractal dimension D provides a measure of surface complexity, describing how the exposed surface area (S) scales with a characteristic linear dimension (L), such that S is proportional to L^D^. For an ideal, smooth surface *D* ≈ 2 (area scales with *L*^2^—for example, 4πR^2^ for a nonporous sphere of radius R), whereas increasingly rough or highly porous solids approach *D* ≈ 3, at which limit the surface area would scale with the sample volume (i.e., ~*L*^3^). Thus, the fractal dimension (D) is a metric that captures the transition from planar surfaces to extremely rough ones [38].

The tortuosity factor (τ) aggregates the deviations of actual transport paths from the straight-line distance into a single dimensionless parameter. Larger values of τ indicate more convoluted pathways and correspondingly greater resistance to diffusive or advective transport. Typical values for porous media range from 2 (e.g., relatively straight, non-intersecting cylindrical pores) to ~7 for highly tortuous pore networks.

#### 2.2.5. Microstructure

For optical microscopy imaging, a high-precision VHX 7000 digital microscope from Keyence (Mechelen, Belgium) with a 1/1.7-inch, 12.22-megapixel CMOS sensor was used. This microscope captured high-resolution 4 K images at full depth of field, at various angles, with a maximum magnification of up to 6000×, allowing for automatic magnification change without the need for lens changes. Tests were conducted on properly prepared samples at magnifications from 200 to 2000 in reflected light.

The chemical composition and surface morphology of the test materials were determined using a Scios 2 LoVac ultra-high-resolution analytical scanning electron microscope (FIB-SEM) (Thermo Fisher Scientific, Waltham, MA, USA) equipped with an UltraDry Premium EDS chemical composition analyzer from Thermo Fisher Scientific. The microscope was equipped with a Schottky cathode and a NICol ultra-high resolution (UHR) electron column in non-immersion mode. The dried samples were attached to aluminum holders using carbon tape and sputtered with carbon to obtain additional conductive layers. Surface morphology studies were carried out under high vacuum conditions using a special Thermo Scientific Trinity detection system designed for simultaneous angular image registration and energy-selective imaging of SE secondary electrons (ETD detector) and BSE backscattered electrons (T1 detector) at an accelerating voltage of 10 keV and a beam current of 1.6 nA. The chemical composition was analyzed at an accelerating voltage of 10 keV and a beam current of 1.6 nA, which enabled a higher number of counts and improved chemical analysis.

## 3. Results

The experimental program provided data on the behavior of mortar composites incorporating fine recycled aggregate and modified with two different types of air-entraining components: a conventional air-entraining admixture (AEA) and preformed polymeric microspheres (PPM). The influence of these modifications was evaluated in terms of fresh-state properties (consistency), mechanical performance (flexural and compressive strength), pore structure characteristics, and resistance to cyclic freezing and thawing.

The reference mixture (REF) served as the baseline for comparison, enabling a clear assessment of the effects introduced by each air-entraining strategy. Special attention was given to the interrelation between strength development and the evolution of pore structure, as well as microstructural observations, to explain the mechanisms governing frost resistance in low-clinker mortars containing fine recycled aggregate. This approach enabled the evaluation of both the initial efficiency and long-term stability of the air–void systems formed by the two types of admixtures.

### 3.1. Consistency and Density

At five min after mixing, both air-entraining admixture (AEA) and preformed polymeric microspheres (PPM) increased the flowability of the mortars compared to the reference mixture (REF). The effect was more pronounced in the mix containing AEA, which showed the largest initial flow diameter. This initial increase in consistency can be attributed to the surface-active action of the air-entraining admixture, which temporarily improves particle dispersion and reduces internal friction within the mixture.

**Figure 4:**
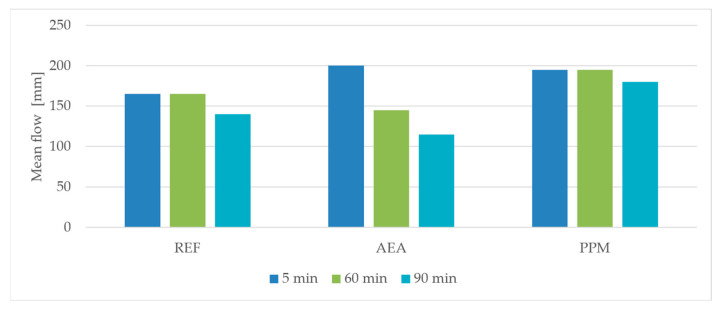
Time-dependent changes in the flow diameter of mortars.

Over time, the flowability of all mixtures decreased, a typical phenomenon associated with the high water absorption of fine recycled aggregate, resulting in gradual water uptake from the paste phase. However, the rate of consistency loss was highest in the AEA-modified mixture. The initial fluidizing effect of the air-entraining admixture was short-lived, and after 60 min it became negligible. After 90 min, the flow diameter of the AEA mixture was even lower than that of the reference mortar (Figure 4). The pronounced decrease in workability observed in the AEA-modified mixture after 90 min can be attributed to several concurrent mechanisms. First, the fine recycled aggregate used in the mixtures exhibits a high water absorption capacity, which progressively reduces the amount of free water available in the paste during extended mixing. Second, the presence of residual unburnt carbon in the fly ash promotes the adsorption of surfactant molecules from the AEA, effectively decreasing the stabilizing capacity of the admixture over time. As a result, the initially formed air–void system becomes progressively destabilized, leading to coalescence and collapse of entrained bubbles. This loss of effective air content directly contributes to the rapid decrease in flowability observed after prolonged mixing.

In contrast, the PPM-modified mixture showed a minor initial increase in flowability. Still, this effect was more stable over time, and its consistency after 60 and 90 min remained higher than that of the reference mixture.

The results of the bulk density measurements for both fresh and hardened mortars (at 28 and 56 days) are presented collectively in Table 2, allowing for a direct comparison of the influence of each admixture on the density evolution of the composites.

### 3.2. Flexural and Compressive Strength

**Figure 5:**
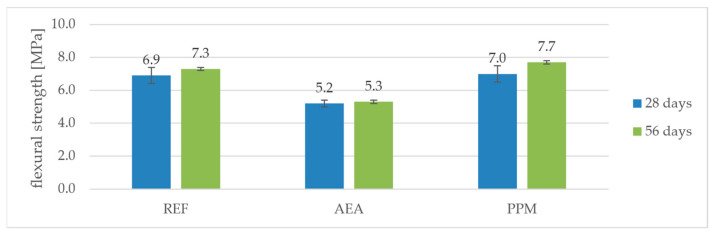
Flexural strength’s dependence on time and composition.

**Figure 6:**
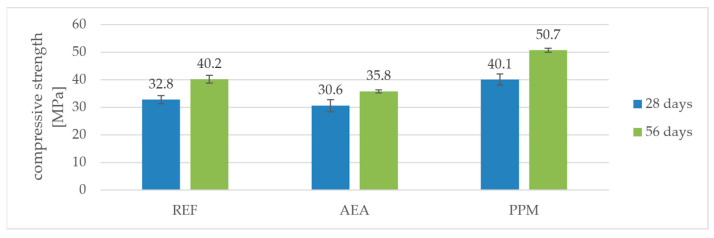
Compressive strength’s dependence on time and composition.

As expected, the introduction of a conventional air-entraining admixture (AEA) resulted in a reduction in both flexural and compressive strength compared to the reference mortar (REF). After 28 days, the flexural strength of the AEA mixture was 5.2 MPa, which is about 25% lower than the 6.9 MPa measured for REF. A similar trend was observed after 56 days: 5.3 MPa for AEA versus 7.3 MPa for REF (a decrease of 27%). The compressive strength of AEA was also lower—30.6 MPa at 28 days and 35.8 MPa at 56 days, compared to 32.8 MPa and 40.2 MPa for REF. This represents a drop of approximately 7% at 28 days and about 11% at 56 days, confirming the well-known effect of air entrainment on reducing the mechanical performance of cementitious composites (Figure 5 and Figure 6).

In contrast, the mixture modified with preformed polymeric microspheres (PPM) exhibited an apparent increase in strength relative to REF. After 28 days, its flexural strength reached 7.0 MPa (about +1% compared to 6.9 MPa for REF), and compressive strength reached 40.1 MPa (+22% compared to 32.8 MPa for REF). After 56 days, the flexural and compressive strengths of PPM further increased to 7.7 MPa and 50.7 MPa, respectively, which corresponds to +6% (flexural) and +26% (compressive) relative to REF at the same age.

When comparing the strength gain between 28 and 56 days, the effect of PPM becomes even more evident. The flexural strength of REF increased by about 6% (from 6.9 to 7.3 MPa), while for AEA the increase was only about 2% (from 5.2 to 5.3 MPa). In contrast, the PPM mixture showed a 10% increase (from 7.0 to 7.7 MPa). A similar trend was observed for compressive strength: the gain was 23% for REF (from 32.8 to 40.2 MPa), 17% for AEA (from 30.6 to 35.8 MPa), and as much as 26% for PPM (from 40.1 to 50.7 MPa).

This indicates that in the presence of polymeric microspheres, strength development continued more intensively between 28 and 56 days, likely due to the enhanced workability and compaction of the fresh mixture, which resulted in a denser microstructure capable of further strength development during prolonged hydration.

A noteworthy observation is that, in mixtures containing PPM, a reduction in volumetric density was accompanied by an increase in compressive strength before freeze–thaw exposure, indicating that the PPM-induced pore system did not weaken the matrix but instead contributed to its densification. In contrast, the AEA-modified mixtures followed the typical trend in which a decrease in density is directly associated with a decline in compressive strength, reflecting the conventional relationship between increased air content and reduced load-bearing capacity.

### 3.3. Freeze-Frost Resistance

The results of the freeze–thaw tests (Table 3 and Table 4) clearly showed that the reference mixture (REF) without any air-entraining component exhibited feeble resistance to cyclic freezing and thawing. After only 25 cycles (Table 3), the flexural strength decreased from 6.0 ± 0.6 MPa to 1.2 ± 0.1 MPa, corresponding to a loss of about 80%, while the compressive strength dropped from 36.9 ± 1.7 MPa to 21.8 ± 1.8 MPa (−40.9%). Although the mass loss was relatively low (−0.50%), the severe decrease in mechanical strength indicates extensive internal damage, confirming that this composition does not meet the F25 frost resistance criterion and would not be suitable for use under freeze–thaw exposure (Table 3).

In contrast, the mixture modified with the conventional air-entraining admixture (AEA) showed high frost resistance. After 25 cycles (Table 3), the flexural and compressive strength losses were only −5.2% and −5.9%, respectively (from 5.8 ± 0.3 MPa to 5.5 ± 0.1 MPa and from 30.7 ± 0.9 MPa to 28.9 ± 2.1 MPa). The mass change was also minimal (−0.20%). Even after 100 cycles (Table 4), the losses remained low, with flexural strength decreasing from 5.9 ± 0.2 MPa to 5.3 ± 0.1 MPa (−10.2%) and compressive strength from 36.9 ± 2.4 MPa to 34.6 ± 1.5 MPa (−6.2%), accompanied by only −1.52% mass loss. These results confirm that the AEA-modified mixture meets the F25 and F100 frost resistance requirements and maintains its mechanical integrity during prolonged freeze–thaw exposure (Table 3 and Table 4).

The behavior of the mixture containing preformed polymeric microspheres (PPM) was markedly different. After 25 cycles (Table 3), the material still retained high strength, with losses of only −12.2% in flexural strength (from 7.4 ± 0.3 MPa to 6.5 ± 0.1 MPa) and −3.4% in compressive strength (from 41.1 ± 2.4 MPa to 39.7 ± 1.8 MPa), and a negligible mass change (−0.27%). This initially suggested that the PPM addition could provide effective freeze–thaw protection. However, after 100 cycles (Table 3), the PPM specimens exhibited catastrophic degradation: flexural strength dropped from 7.6 ± 0.1 MPa to 1.9 ± 0.1 MPa (−75%), and compressive strength from 50.6 ± 0.6 MPa to 33.0 ± 1.8 MPa (−34.8%), even though mass loss remained very low (−0.01%) (Table 3 and Table 4). These results demonstrate that although PPM admixture initially provided frost resistance (F25), it failed to ensure long-term durability (F100).

Interestingly, the loss of performance in the PPM mixture occurs between approximately 40 and 56 days of curing, which coincides with the time when the continued hydration of the low-clinker binder may begin to fill the initially formed voids after partial degradation of the polymeric spheres. As a result, the protective pore system progressively disappears, and the material becomes vulnerable to freeze–thaw damage.

It was also observed that early signs of degradation were more clearly reflected in the flexural strength results than in compressive strength. This is likely because flexural strength is more sensitive to the formation of microcracks across the cross-section, and thus can serve as an early indicator of frost-induced damage development in cementitious composites.

### 3.4. Porosity

**Figure 7:**
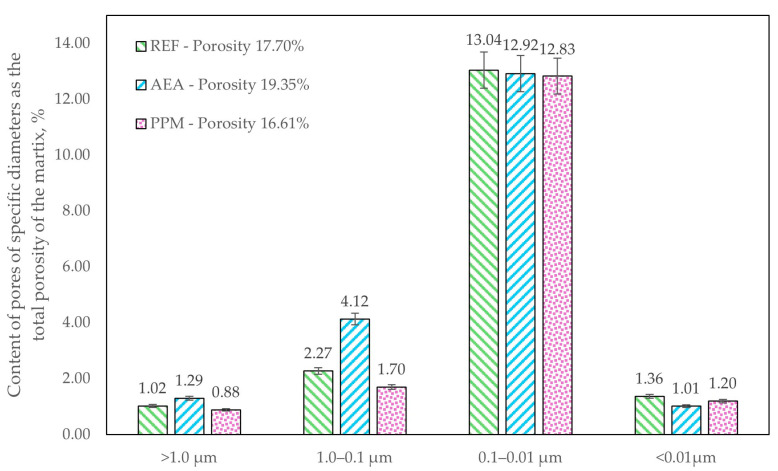
Total porosity of investigated cementitious composites and total volumetric content of pores of specific diameters.

The pore-network characteristics differed between the investigated series. For the analysis, four pore-size classes were defined: <10 nm (gel pores associated with the C-S-H matrix), 10–100 nm (pores associated with crystallized binder-hydration products), 100–1000 nm (capillary pores), and >1000 nm (macropores) [39]. The studied admixtures contributed to distinct effects on both total porosity and pore-size distribution (Figure 7). The reference composite was characterized with a total porosity of 17.70%. Addition of an air-entraining agent (AEA) increased total porosity to 19.35%, whereas incorporation of polymeric microspheres (PPM) reduced total porosity to 16.61%. Changes in the volumetric fraction of specific pore-size classes were most pronounced in the capillary range (100–1000 nm). In the AEA-modified series, the volumetric fraction of 100–1000 nm pores increased to 4.37% (from 2.48% in the reference), whereas in the PPM-modified series, it decreased to 1.50%—an approximately 40% reduction relative to the reference, suggesting a densification of crystallized hydration products within the cement matrix.

**Figure 8:**
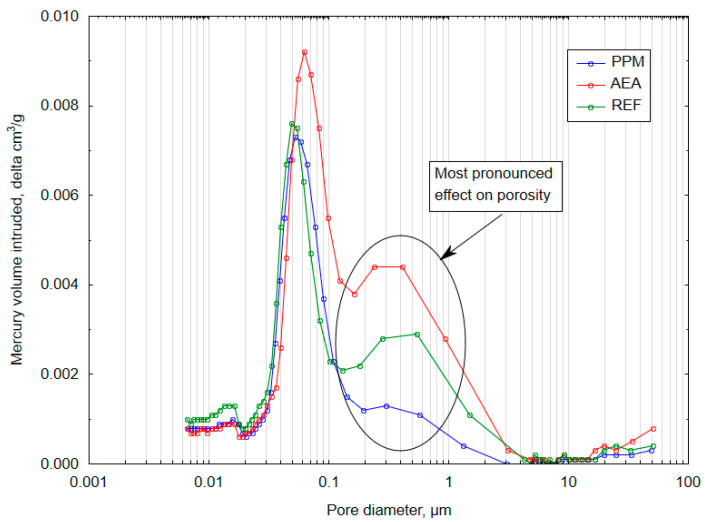
Volumetric pore diameter distribution of investigated cementitious composites.

**Figure 9:**
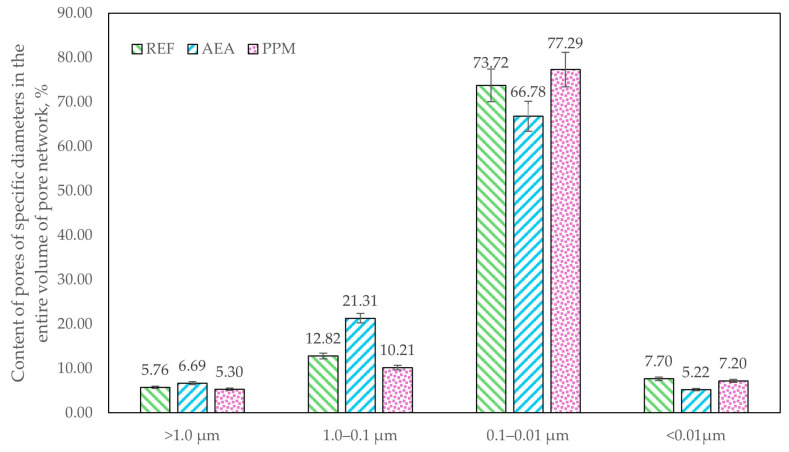
Distribution of pores in specific diameter ranges in the entire volume of the pore network.

Although total volumetric porosity was comparable across all series, the distribution of pores by diameter differed markedly (Figure 8 and Figure 9). The most pronounced changes occurred in the 100–1000 nm pore diameter range. In the AEA-modified series, 21.31% of all pores fell within this capillary range (approx. 66% increase relative to the reference), indicating a detrimental influence of the admixture on the hydration process and a lower amount of crystallized hydration products. Conversely, in the PPM-modified series, the share of pores in this range decreased to 10.21% (approx. 20% reduction compared to the reference), indicating a different, PPM-linked influence on the crystallization pathway and resulting refinement of the pore structure.

**Figure 10:**
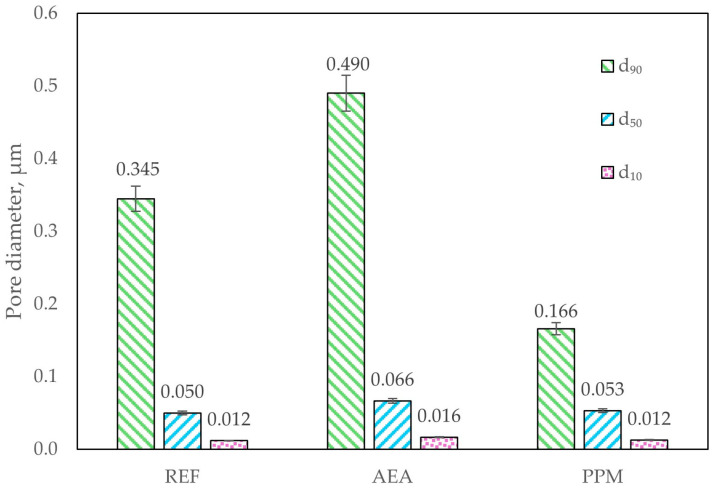
Pore size characteristics of the composite (d_90_—90% of pore volume consists of pores below the given diameter; d_50_—50% of pore volume consists of pores below the given diameter, d_10_—10% of pore volume consists of pores below the given diameter).

The applied modifications resulted in opposing alterations to the pore characteristics of the composites (Figure 10). These changes were reflected in the 90th-, 50th- and 10th-percentile pore diameter, which represents the pore diameter below which x% of the pore volume is contained. Incorporation of the air-entraining agent (AEA) shifted the pore-size distribution toward larger voids, increasing d_90_ to 0.490 µm from 0.345 µm in the reference series (approx. 42% increase). In contrast, the addition of polymeric microspheres (PPM) contributed to the refinement of the pore network, reducing d_90_ to 0.166 µm (approx. 52% decrease relative to the reference). It can be concluded that AEA promoted the formation of larger pores, whereas PPM contributed to an overall densification of the cement matrix.

### 3.5. Microstructure

**Figure 11:**
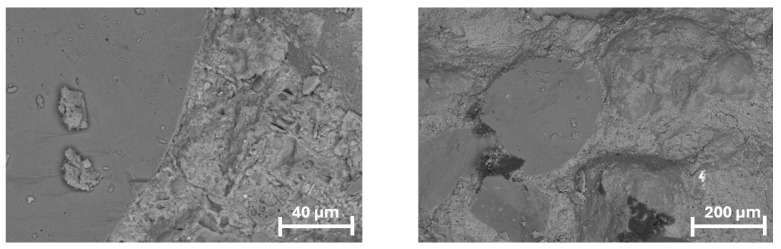
Microstructure of the reference mortar (REF) observed under SEM in BSE mode: (**left**)—200× magnification, providing an overview of the aggregate–matrix interface; (**right**)—2000× magnification, showing a dense interfacial transition zone (ITZ) with no evidence of increased porosity or microcracking.

In the reference specimen (REF), the interfacial transition zone (ITZ) between the cement matrix and the aggregate appeared well-consolidated and free from microstructural disturbances. No signs of increased porosity or microcracking were observed along the aggregate boundary, indicating a stable bond and uniform hydration products. The microstructure in this area was dense, with no evidence of secondary voids or defects, which suggests adequate compaction and the absence of internal stress concentrations at the matrix–aggregate interface (Figure 11).

**Figure 12:**
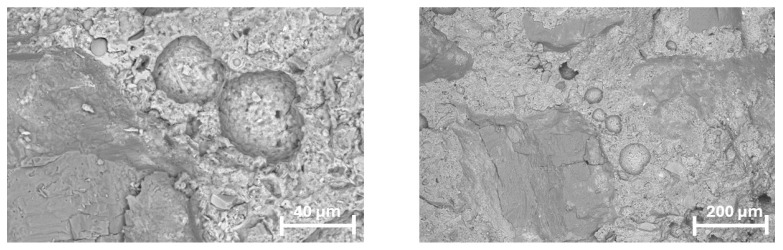
Microstructure of the AEA-modified mortar observed under SEM in BSE mode: (**left**)—200× magnification, showing increased pore concentration near recycled aggregate grains; (**right**)—2000× magnification, revealing uniformly sized entrained air voids within the cement matrix.

In the AEA-modified mortar, a pronounced increase in the number of air voids can be observed within the cement matrix. The concentration of pores is particularly noticeable in the vicinity of recycled aggregate particles, indicating intensified void formation within the interfacial transition zones (ITZ). Despite the higher porosity, the voids display relatively uniform size and distribution, which suggests that the air-entraining admixture effectively generated a controlled air–void system typical of air-entrained composites (Figure 12).

**Figure 13:**
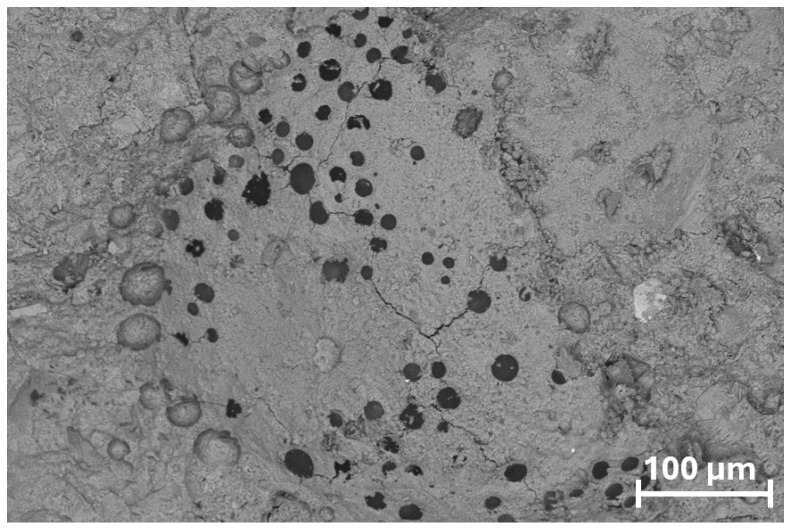
Microstructure of the PPM-modified mortar observed under SEM in BSE mode—300× magnification.

In the mortar containing preformed polymeric microspheres (PPM), the microstructure reveals partially filled spherical cavities corresponding to the locations of the polymer spheres. Although these voids are still visible, many of them appear partially occluded or lined with hydration products, indicating progressive infilling over time. Furthermore, certain cavities are associated with areas where recycled aggregate particles detached, leaving behind smooth, rounded voids rather than open pore spaces (Figure 13).

This microstructural evidence explains the loss of frost resistance at later ages: while the initial void system created by the polymer spheres may have contributed to improved early properties, the gradual reduction of effective air voids through continued hydration prevents the formation of a stable freeze–thaw protection mechanism.

**Figure 14:**
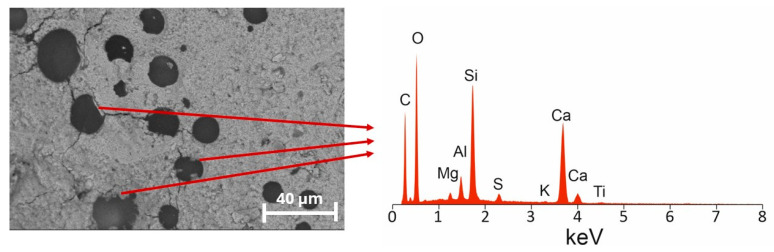
EDS mapping at the rim of a former polymeric pore showing C–S–H phases growing into the interior of the sphere, indicating the progressive infilling of the void due to prolonged hydration.

The EDS analysis conducted at the edge of the former polymeric sphere, in the area where calcium–silicate–hydrate (C–S–H) phases are visibly growing into the interior of the sphere, revealed a distinct presence of these hydration products. This indicates that hydration progressed beyond the durability of the polymer shell, allowing hydration products to penetrate and partially fill the originally hollow pore (Figure 14).

The degradation or rupture of the polymeric membrane enabled the ingress of hydration products, resulting in the progressive occlusion of the intended air void. As a consequence, the pore ceased to function as an effective pressure-relief space during freeze–thaw cycling, which explains the loss of frost resistance observed at later testing ages. This microstructural evidence confirms that in low-clinker systems with prolonged hydration, preformed polymeric pores may not remain stable in the long term, despite their initial effectiveness in improving fresh consistency and early-age performance.

### 3.6. Preliminary Cost Analysis

A simplified economic analysis compared the cost of polymeric microspheres (PPM) with that of a conventional air-entraining admixture (AEA). The evaluation reveals that PPM is several times more expensive than AEA, and at the required dosage, it results in a substantially higher admixture cost per cubic meter of mortar. In contrast, AEAs, even at slightly higher dosages, remain considerably more economical, resulting in a much lower overall cost contribution. Given that PPM did not provide sufficient long-term freeze–thaw resistance in the tested low-clinker system, its significantly higher cost, combined with the lack of clear performance benefits, may limit its practical suitability for construction applications.

## 4. Discussion

Durability considerations, particularly resistance to cyclic freeze–thaw, are central to the development of sustainable cementitious composites [23,40]. This study addressed that challenge by combining two contemporary strategies: the use of low-clinker binders (high replacement levels of fly ash and GGBFS) to reduce embodied carbon of the composite, and the incorporation of fine recycled aggregate to promote circularity within the construction sector. Both strategies, however, alter hydration kinetics and microstructure in ways that can compromise early-age performance and frost resistance [41,42]. The present work evaluated two contrasting approaches to introducing protective voids into such systems: a conventional air-entraining agent (AEA), which relies on the in situ generation and stabilization of air voids, and preformed polymeric microspheres (PPM), which introduce discrete, dimensionally stable voids.

In the experimental program, the effects of the air-entraining agent (AEA) on the performance of low-clinker composites were broadly consistent with its well-documented behavior in conventional Portland-cement systems [43]. AEAs, typically liquid admixtures added during batching, function by stabilizing dispersed gas bubbles within the fresh paste. These discrete voids persist through setting and hardening of cement matrix, serving as internal pressure-relief sites. The practical consequence is a marked improvement in resistance to damage from cyclic freezing and thawing, owing to the mitigation of hydraulic pressures that would otherwise generate microcracking in a saturated paste. From a compositional standpoint, most commercial AEAs are based on surface-active agents (historically wood-resin salts or rosins and, more recently, synthetic surfactants) that lower interfacial tension and promote the formation and stabilization of a fine air–void system. During testing, AEA addition produced the expected microstructural signature: an increase in total porosity accompanied by a redistribution of pore sizes toward larger capillary pores. This microstructural modification correlated with improved freeze–thaw durability, but also with a measurable reduction in compressive strength-a well-recognized trade-off in which increasing the entrained air content reduces the apparent density of the composite.

In low-clinker systems incorporating supplementary cementitious materials (SCMs) such as fly ash and GGBFS, additional complexities can arise. Adsorptive interactions between AEAs and particle surfaces—particularly unburnt carbon in some fly ashes—can diminish surfactant availability and necessitate higher dosages to achieve an equivalent air–void system (in the conducted research, 1.5% m.b., compared to 0.2% m.b., maximum dosage recommended by the manufacturer). Consequently, attaining durable and mechanically efficient low-clinker concretes with AEAs requires not only control of dosing but also consideration of SCM characteristics, mixing procedures, and the evolving pore structure as hydration progresses.

To address these limitations, the present study investigated whether chemically generated air–void systems produced by conventional air-entraining admixtures (AEAs) can be replaced with a purely physical approach: the incorporation of preformed polymeric microspheres (PPMs). Unlike AEAs, which rely on surfactant-mediated air void generation and are susceptible to adsorption by supplementary cementitious materials, PPMs introduce dimensionally stable inclusions that do not depend on in situ foaming or on the continued availability of surface-active molecules.

**Figure 15:**
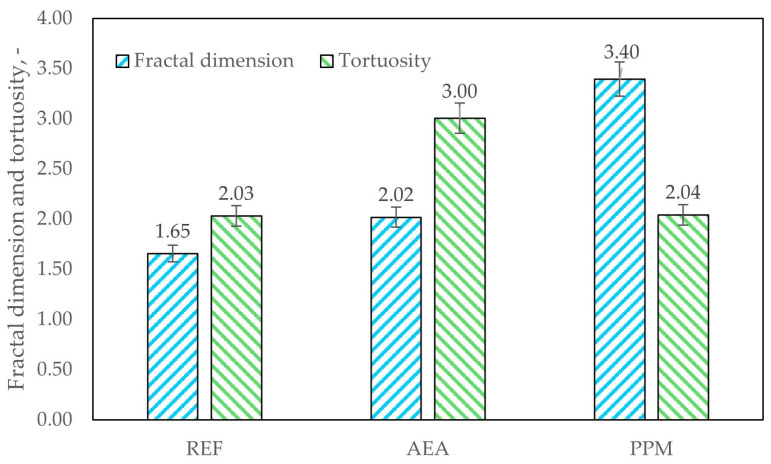
Fractal dimension and tortuosity of investigated pore networks.

The experimental evidence supports and clarifies the hypothesized advantages of a PPM-based void system, while also highlighting important limits and phenomena linked with PPM presence in the cement matrix. SEM imaging showed extensive crystallization of hydration products within and around the voids introduced by polymeric microspheres: rather than remaining as inert, free cavities, many PPM-induced pores became sites of intensive crystal growth, producing dense masses of hydration products that bridged and occluded portions of the original void space. Mercury-intrusion porosimetry corroborated this microstructural evolution, showing a measurable reduction in total porosity and a clear refinement of the pore-size distribution toward finer capillary pores; importantly, the tortuosity of the transport pathways remained comparable to that of the reference material, indicating that pore refinement did not substantially increase the geometric complexity of connected flow paths (Figure 15). The crystallization observed in SEM was quantitatively reflected by an increase in the pore-system fractal dimension from 2.03 (reference) to 3.40 (PPM-modified), consistent with a more irregular, space-filling internal structure produced by extensive crystal growth on the surface of PPM-induced voids.

These microstructural changes had mechanical and durability consequences. Compressive strength test showed a substantial improvement for PPM-modified composites: at 28 days, the reference compressive strength was approx. 32.8 MPa compared with 40.1 MPa for the PPM series (approx. 22.3% increase), and at 56 days, the strengths were 40.2 MPa (reference) and 50.7 MPa (PPM) (approx. 26.1% increase). Such gains are consistent with the mercury porosimetry and SEM observations: a reduction in total porosity and intrapore crystallization both contribute to a denser, load-bearing cementitious matrix. In freeze–thaw testing, PPM modification produced a clear improvement at short–to–moderate cycling: the PPM-modified specimens passed 25 cycles, whereas the reference failed under the same test protocol (the reference composite failed, with a reduction in compressive strength above 20%, at 25 and 100 cycles). However, PPM specimens did not pass the extended 100-cycle test and ultimately failed at that level of exposure.

Taken together, the studies conducted indicate that PPMs can produce a physically stable, strength-enhancing refinement of the pore structure in low-clinker, SCM-containing mortars, validating several of the hypothesized benefits, including a mitigated strength penalty relative to conventional AEA systems. At the same time, the tendency for hydration products to crystallize within PPM-generated voids can progressively reduce the effective pressure-relief capacity of the inclusions, which likely explains why PPMs improved short-term freeze–thaw performance but did not provide sufficient protection under extended cycling. However, considering the effect of PPMs on the porosity of the composite, the authors intend to investigate whether this effect can be mitigated through optimization of the composite’s composition and an increase in the PPM dosage.

## 5. Conclusions

The study conducted demonstrated that achieving reliable freeze–thaw resistance in low-clinker mortars containing fine recycled aggregate remains a major challenge. The use of fly ash and GGBFS significantly modifies hydration kinetics and pore development, making it difficult to obtain a stable and long-lasting air–void system using conventional approaches. The AEA-modified mixture required a high dosage to generate a sufficient void system, yet this produced considerable variability in consistency, with flow diameter after 90 min dropping below that of the reference mixture. This instability is a critical limitation for practical applications, especially when mixtures must retain workability during transport and placement. Therefore, the performance of traditional air-entraining agents in low-clinker composites is strongly dependent on SCM characteristics and mixing time.

Preformed polymeric microspheres (PPM) initially appeared to be promising alternatives for improving both rheological and mechanical performance. Their incorporation did not lead to rapid workability loss, and they increased compressive strength at 28 days from 32.8 MPa to 40.1 MPa and at 56 days from 40.2 MPa to 50.7 MPa. Mercury intrusion porosimetry confirmed a refinement of the pore structure in the PPM mixture, with total porosity reduced to 16.61% compared to 17.70% in the reference mixture and 19.35% in the AEA-modified mixture. The pore-size distribution shifted toward finer voids, with d90 decreasing from 0.345 μm to 0.166 μm. These results indicate that polymeric microspheres enhance matrix densification and promote the development of a refined microstructure.

Despite these improvements, PPM modification did not ensure long-term freeze–thaw durability. After 25 cycles, the PPM mortar still exhibited low strength losses, performing similarly to the AEA mixture at this stage. After 100 cycles, however, PPM specimens experienced a drastic reduction in flexural and compressive strength, even though mass loss remained negligible. SEM observations showed extensive growth of hydration products inside the residual voids of degraded polymer spheres, confirming the gradual disappearance of the originally protective pore system. This mechanism aligns with the slow hydration of low-clinker binders, which promotes progressive infilling of artificial voids and diminishes their function as pressure-relief sites during freezing.

Therefore, although early-age tests may suggest that polymeric microspheres provide adequate frost protection, their stabilizing effect does not persist once hydration advances and the PPM-derived cavities become partially or fully occluded. In contrast, the AEA-modified mortar retained frost resistance up to 100 cycles, with compressive strength loss limited to −6.2%. These findings indicate that ensuring long-term frost resistance in low-clinker eco-concretes requires solutions capable of maintaining pore functionality throughout prolonged hydration. Future research should explore optimized PPM dosages, modified polymer chemistries with improved durability, hybrid approaches combining PPM with AEA, and the influence of binder composition on the stability of preformed void systems.

## Figures and Tables

**Table 1:** Mass proportions of the tested mortar mixtures (per 1 m^3^ of fresh mortar).

Mix ID	REF	AEA	PPM
Cement, kg	511	511	511
Water, kg	256	248	256
Natural sand, kg	1282	1282	1282
Fine Recycled aggregate, kg	230	230	230
Air-entraining admixture, kg	0	7.67	0
Polymeric microspheres, kg	0	0	6

**Table 2:** Volume density of fresh mortar and hardened specimens after 28 and 56 days of curing.

Mix ID	REF	AEA	PPM
Volume density of fresh mortar after 5 min of mixing, g/cm^3^	2.21	1.98	2.20
Volume density of fresh mortar after 90 min of mixing, g/cm^3^	2.21	1.97	2.20
Volume density after 28 days, g/cm^3^	2.07	1.88	2.10
Volume density after 56 days, g/cm^3^	2.08	1.88	2.09

**Table 3:** Mechanical properties and mass loss of mortars after 25 freeze–thaw cycles.

	Flexural Strength			Compressive Strength			Mass Difference, %
	Control, MPa	Tested, MPa	Difference, %	Control, MPa	Tested, MPa	Difference, %	
REF	6.0 ± 0.6	1.2 ± 0.1	−80.0	36.9 ± 1.7	21.8 ± 1.8	−40.9	−0.50
AEA	5.8 ± 0.3	5.5 ± 0.1	−5.2	30.7 ± 0.9	28.9 ± 2.1	−5.9	−0.20
PPM	7.4 ± 0.3	6.5 ± 0.1	−12.2	41.1 ± 2.4	39.7 ± 1.8	−3.4	−0.27

**Table 4:** Mechanical properties and mass loss of mortars after 100 freeze–thaw cycles.

	Flexural Strength			Compressive Strength			Mass Difference, %
	Control, MPa	Tested, MPa	Difference, %	Control, MPa	Tested, MPa	Difference, %	
REF	Degradation after 75 cycles						
AEA	5.9 ± 0.2	5.3 ± 0.1	−10.2	36.9 ± 2.4	34.6 ± 1.5	−6.2	−1.52
PPM	7.6 ± 0.1	1.9 ± 0.1	−75.0	50.6 ± 0.6	33.0 ± 1.8	−34.8	−0.01

## Data Availability

The original contributions presented in the study are included in the article, further inquiries can be directed to the author.
